# Sacrococcygeal teratoma in one twin: a case report and literature review

**DOI:** 10.1186/s12884-020-03454-1

**Published:** 2020-12-02

**Authors:** Qing Hu, Yiyun Yan, Hua Liao, Hongyan Liu, Haiyan Yu, Fumin Zhao

**Affiliations:** 1 Department of Obstetrics and Gynecology, West China Second University Hospital, Sichuan University, Chengdu, China; 2 Key Laboratory of Birth Defects and Related Diseases of Women and Children (Sichuan University), Ministry of Education, Chengdu, China; 3 Department of Radiology, West China Second University Hospital, Sichuan University, Chengdu, China

**Keywords:** Twin pregnancy, fetal sacrococcygeal teratoma, literature review

## Abstract

**Background:**

Sacrococcygeal teratoma is one of the most common congenital tumors in newborns and infancy. The incidence is 1 per 20,000–40,000 live births. Ultrasonography is an optimal method for prenatal screening and diagnosis of fetal sacrococcygeal teratoma. MRI can be used to assist in the diagnosis. However, sacrococcygeal teratoma in the twin pregnancy is rare.

**Case presentation:**

We reported a case of one twin with sacrococcygeal teratoma in dichorionic-diamniotic twin pregnancy.One twin with sacrococcygeal teratoma was diagnosed at the second trimester by ultrasonic examination and another twin was normal. A regular and careful antenatal care was conducted by the multidisciplinary team. The parents refused to perform the fetal MRI and examine the chromosome of both twin.At 37 + 1 of gestation, planned cesarean section was performed. The healthy male co-twin (twin A) weighed 2880 g.The male twin with SCT (twin B) weighed 2900 g, complying with 6 × 3 × 3 cm cystic and solid mass in sacrococcygeal region. At four days of age twin B underwent excisional surgery of the sacrococcygeal teratoma and coccyx and discharged 7 days after surgery. The mother and both babies were followed up and are all in good health until now.

**Conclusion(s):**

Sacrococcygeal teratoma in twin pregnancy is rare. Early antenatal diagnosis is important. Once the sacrococcygeal teratoma is diagnosed, clinicians should be aware of the associated maternal and fetal complications. Expecting parents should be counseled by the multidisciplinary team about the management and prognosis of the STC twin and co-twin. Prompt surgical excision of the sacrococcygeal teratoma after birth should be suggested.

## Background

Teratoma originates from early embryonic pluripotent stem cells, and the Hensen’s node in front of the coccyx is the site where pluripotent stem cells are concentrated. Therefore, sacrococcygeal teratoma(SCT) is the most common tumor found in newborns and infants with the incidence of 1 per 20,000 ~ 40,000 live births [1, 2].The morbidity and mortality associated with SCT may be associated with dystocia associated with tumor masses, preterm birth secondary to excessive dilatation of the uterus caused by polyhydramnios, and fetal development, edema caused by fetal anemia and / or high output heart failure secondary to arteriovenous steal in the tumor mass. It is worth noting that the prognosis has nothing to do with the size of the tumor. However, the prognosis of parenchyma vascular masses is worse than that of cystic masses [2]. Fetal surgery has been shown to be a treatment of fetal SCT by in utero resection to improve the fetal outcome. Most reported fetal sacrococcygeal teratoma was singleton pregnancy, while, the rate of one twin with sacrococcygeal teratoma is very rare and no consensus in fetal SCT resection during pregnancy. We reported a case of one twin with SCT in dichorionic diamniotic twin pregnancy and the timely intervention allowed the survival of both twins. Additionally, we used a list of keywords including “sacrococcygeal teratoma”, “twin pregnancy” and “multiple pregnancy” to perform an extensive search and conducted a literature review in English and Chinese about the perinatal management and postnatal outcomes of twin pregnancies compared with one fetus with prenatally diagnosed sacrococcygeal teratoma.Written informed consent was obtained from the couple before the procedure and manuscript publication. The treatment procedure followed ethical principles, all data were collected from chart reviews, and approval was obtained from the Institutional Review Board.

## Case presentation

**Fig. 1:**
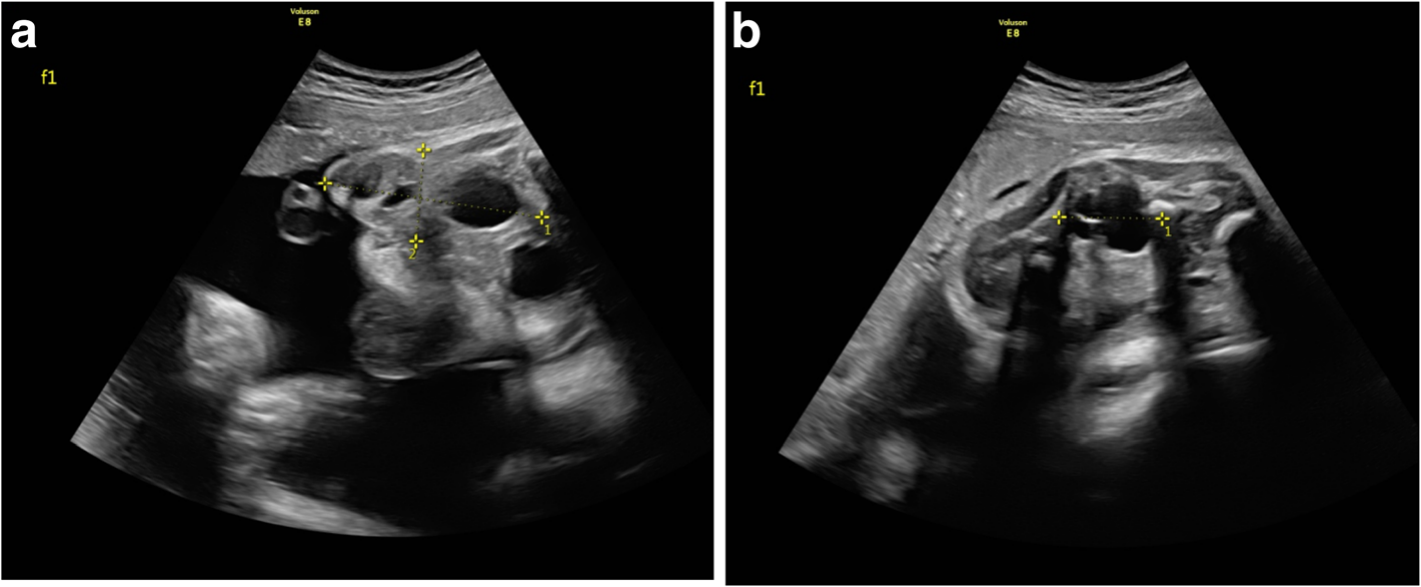
**a, b** Ultrasound view of the SCT in twin B

**Fig. 2:**
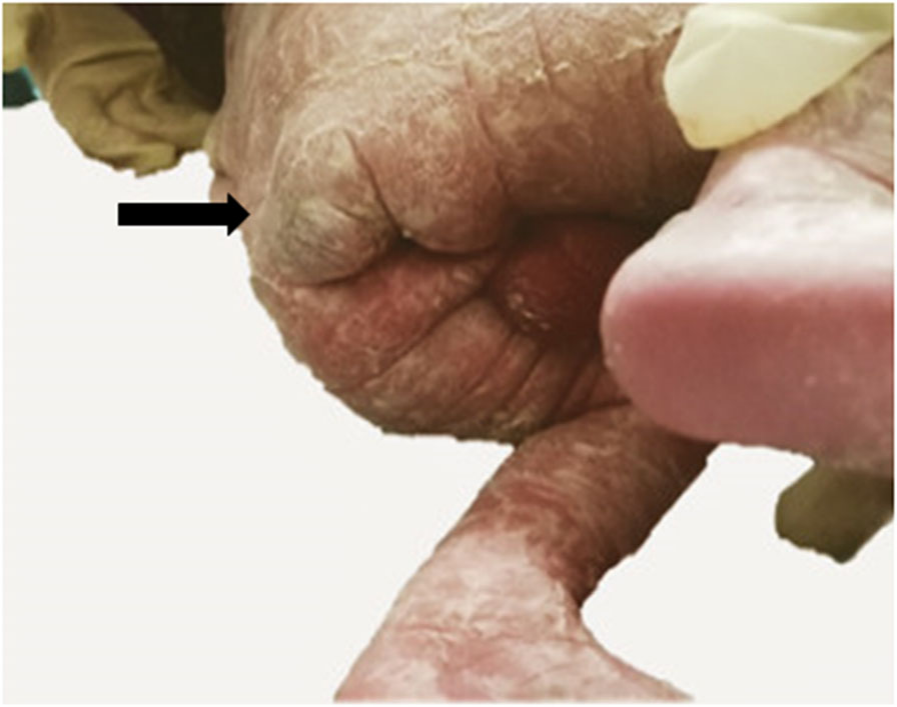
View of the SCT in twin B at birth

**Fig. 3:**
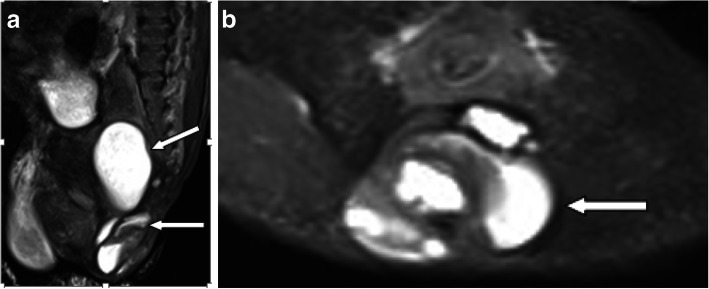
**a**MRI sagittal T2W1 image of the SCT at two days of age. **b** MRI axial T2W1 image of the SCT at two days of age

**Fig. 4:**
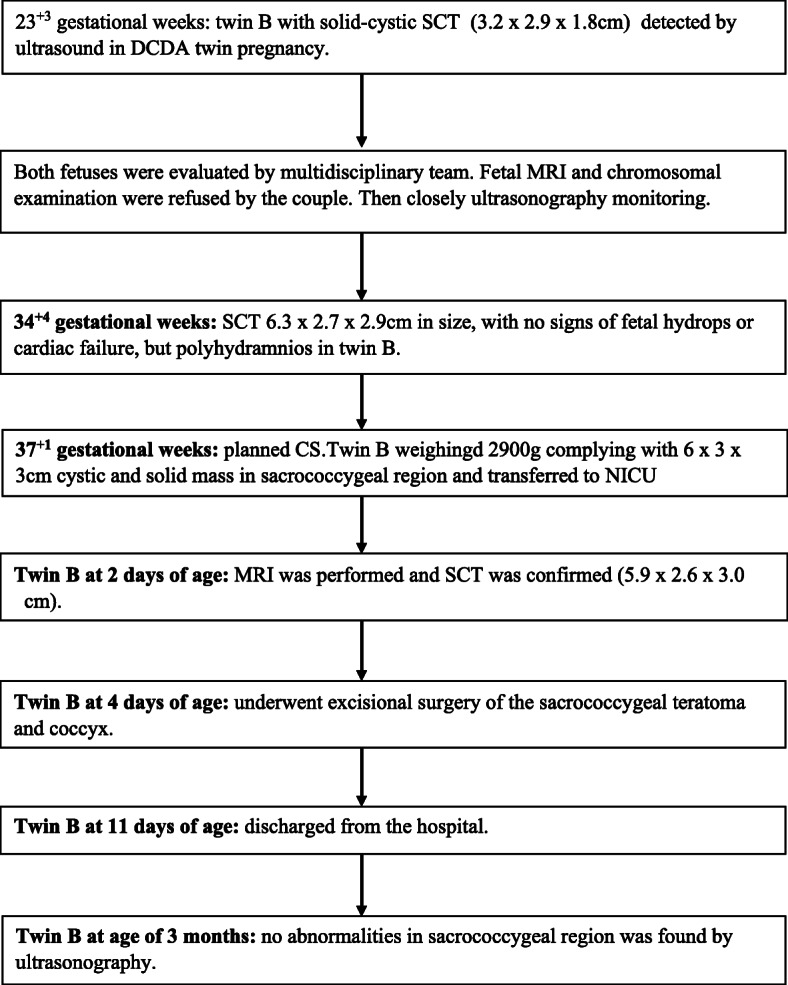
Flow diagram of this case

A 25-year-old woman, gravida 1, para 0, conceived dichorionic diamniotic twin pregnancy spontaneously. The couple was not consanguineous and had no reported history of medication, hereditary disease, substance abuse, or a family history of congenital anomalies and teratoma. The patient’s serology was negative for human immunodeficiency virus (HIV), venereal disease research laboratory (VDRL), and hepatitis B surface antigen (HBsAg) and she had no diabetes mellitus. During a routine second trimester ultrasound at 23 + 3 weeks’ gestation, a 3.2 cm mixed solid and cystic SCT starting from the sacral area was detected in one twin (twin B) with no other fetal abnormalities,and co-twin (twin A) with no abnormality. Given this condition, the patient was transferred to our department. The couple was extensively counseled by the multidisciplinary team regarding the diagnosis, treatment, and prognosis of the SCT twin. The parents refused to perform the fetal Magnetic Resonance Imaging(MRI) and examine the chromosome of both twins.The family opted to continue the pregnancy and the fetuses were followed closely. The gradual growth of the SCT mass was identified by sonography with no signs of hydrops and fetal cardiac failure. At 34 + 4 weeks’ gestation, on follow-up ultrasound, the fetus was detected with polyhydramnios and no signs of hydrops and the solid and cystic mass 6.3 × 2.7 × 2.9 cm (Fig. 1a and b).Planned cesarean section was performed at 37 weeks and 1 day. The healthy male co-twin (twin A) weighed 2880 g with Apgar scores of 10 and 10 at 1 and 5 minutes, respectively. The male twin with SCT (twin B) weighed 2900 g with Apgar scores of 10 and 10 at 1 and 5 minutes, respectively, complying with 6 × 3 × 3 cm cystic and solid mass in sacrococcygeal region (Fig. 2).The neonate transferred to NICU due to SCT. At two days of age, Magnetic Resonance Imaging (MRI) was performed and showed: The sacrococcygeal region occupied by a cystic mass about 5.9 × 2.6 × 3.0 cm, which partly located behind the presacral peritoneum, the upper margin to the superior margin of sacral 3, the lower margin to the inferior margin of the caudal vertebra about 2.6 cm, boundary was not clear, the larger cystic focus is located in the presacral region, and the size is about 2.0 × 3.2 × 1.8 cm, a little T1WI and T2WI high signal can be found inside. The mass pushed the anorectal, rectum to the right front. The anal canal and sacral canal were not suffered (Fig. 3a and b). At four days of age, giant sacrococcygeal teratoma resection, coccyx resection, pelvic floor reconstruction and skin flap plasty were performed. The excision of the coccyx and mass was complete.The histopathology showed mature sacrococcygeal teratoma with negative margins. The baby discharged 7 days after surgery. Both babies and the mother were followed up. At age of 3 months, the baby with SCT removed was evaluated by ultrasonography and no abnormalities in sacrococcygeal region, with no uncontrolled urination, difficult bladder emptying, pyelonephritis and constipation.Both babies are in normal development until now. The flow diagram of this case is shown in in Fig. 4.

## Discussion and conclusions

Sacrococcygeal teratoma is one of the most common congenital tumors in newborns and infancy. It happens in 1 per 20,000 ~ 40,000 births [1, 2] and common in female [2]. Neonatal death rate of prenatal diagnosed SCT is as high as 24% and the dead cases had higher tumor volume index and concentrations of NT-pro-BNP and cTnT than the survivors [3]. The tumor originated from pluripotent cells in the Hensen’s node, which escaped normal induced stimulation [2, 4].

According to Altman’s classification,there are four types of SCT as follows: Type I tumor mainly protruding from the sacrum and coccyx, presenting hip deformation, Type II tumor mainly external, but has larger pelvic components,Type III tumor mainly in the pelvis and small in the lateral mass of the buttocks,Type IV tumor completely internal and has no external components. All four types could have intraspinal nerve damage. The mass of SCT can be cystic, solid, or mixed. According to histopathology, teratoma can be divided into mature teratoma, immature teratoma, mixed teratoma and malignant teratoma. Sacral teratomas are mostly benign.The more cystic the tumor, the more mature it is. Meanwhile, most malignant tumors are solid masses [4]. Usui reported the mortality rates in predominantly cystic tumor component and predominantly solid tumor component were 2% and 33%, respectively [5]. In the present case,it was Type II SCT.

Fetal sacrococcygeal teratomas (SCTs) occur in one to two per 20 000 pregnancies [6]. Prenatal ultrasound graphic examination is useful in the diagnosis of sacrococcygeal teratomas and the main manifestation of fetal sacrococcygeal teratoma is sacral mass. Nevertheless, ultrasound examination may be affected by maternal obesity, amniotic fluid, and fetal position, especially difficult to evaluate the extension of SCT in the pelvic and abdominal cavity, the compression to the pelvic organs or tiny tumor. MRI can be used to assist in diagnosis [2].

Some fetal SCT may rapidly grow and present richly formed blood vessels in the tumor, and even arteriovenous fistula is formed, which results in polyhydramnios, fetal blood loss, fetal high output heart failure, fetal anemia, fetal edema and fetal death. SCT protruding growth can result in fetal bladder obstruction, fetal hydronephrosis, fetal ureteral dilatation, or fetal gastrointestinal obstruction [2–4, 7–9],etc.

Due to the complication of fetal SCT, fetal interventions might be suggested, which includes open fetal surgery to resect the tumors, radiofrequency ablation, major vessel laser ablation, and vessel alcohol sclerosis to prevent the fetal high output cardiac failure, intra-tumor arteriovenous shunt, reduce tumor volume and fetal edema in order to improve the fetal outcomes. Sometimes, amnioreduction should be performed to prevent preterm labor and cyst aspiration should be done to prevent tumor rupture at delivery [5, 9, 10].

When the diameter of fetal SCT is larger than 5 cm, in order to avoid dystocia and birth injury, intertumoral hemorrhage and tumor rupture, cesarean section is suggested at delivery [7]. Benign sacrococcygeal teratoma has a good outcome after early surgery. The occurrence of malignancy in SCT appears to be related to age at presentation and age at resection [11, 12].

The percentage of malignant transformation within 2 months after birth is 20%. 40% after 4 months, so it should be completely removed as soon as possible after birth to prevent malignant transformation. Residual coccyx and tumor rupture during operation are the predominant risk factors for recurrence [13]. Therefore, the tumor and coccyx should be removed as completely as possible during the operation. At present, most professionals thought that for benign teratoma, removing the coccyx and avoiding the residual damage of the tumor cyst wall during operation is the key to prevent recurrence. Other studies had shown that even complete resection of sacrococcygeal teratoma, it can recur many years after initial resection. Therefore the patients should be closely followed up to adulthood [13]. Related studies show that tumor size, proportion of solid components, growth rate, vascular richness, degree of cardiac function damage, fetal edema, polyhydramnios and maternal complications are related to poor fetal prognosis [14, 15]. In addition to the above, the gestational age of delivery is also an independent prognostic factor [5].

Neonatal SCT with high output heart failure, intertumoral hemorrhage and perioperative hemorrhage are the most common causes of early death, which are closely related to the size of the tumor. The mortality caused by neonatal SCT hemorrhage is as high as 3.8%, accounting for nearly 70% [16] of the total neonatal SCT mortality. Fetal SCT is usually reported in singleton pregnancy.Few literatures reported STC in one twin in twin pregnancy. We performed an extensive search and make a literature review in English and Chinese and found fewer than 9 cases of SCT in one twin had been reported [10, 17–22]. Detailed information are showed in Table 1.

**Table 1:** Reported Cases of one twin with SCT

Reference	Cases	Mode of conception	Twin type	Diagnosis GA	tumor component	hydrops fetalis	Polyhydramnios	delivery GA	Mode of delivery	SCT twin			Co-twin		
										BW(g)	Sex	Outcome	BW(g)	Sex	0utcome
Hedrick [10] (2004)	3	No data	No data	No data	No data	No data	No data	No data	No data	No data	No data	No data	No data	No data	No data
Albu [17] (2019)	1	IVF	DA	17wks	solid-cystic	No	Yes	/	/	/	No data	selectively discontinue this twin	No data	F	live
Ayzen [18] (2006)	1	not mention	MCDA	second trimester	solid	Yes 24wks	No	26 + 2 wks	CS	No data	F	Died shortly after birth	600	F	live
Chen [19] (2004)	1	not mention	DCDA	24wks	cystic	No	No	36wks	CS	3052 (SCT10 × 8 × 6 cm)	M	underwent excision of the intrapelvic and extrapelvic teratoma at age 2 days, **live**	2440	F	live
Sherowsky [20] (1985)	1	induction of ovulation with clomiphene citrate	unknown	30wks	cystic and solid	No	Yes	32wks	CS	3280 (SCT 30 × 17 cm)	M	Underwent SCT resection at 3 days of age, **live**	1420	F	live
Zhang [21] (2018)	1	No data	No data	No data	No data	No data	No data	No data	No data	No data	No data	No data	No data	No data	No data
Lou [22] (2009)	1	IVF	DCDA	24wks	cystic and solid	No data	Yes	34 wks	CS	4200 (SCT30 × 25 × 25 cm)	F	Underwent SCT resection in 1 day after birth, died 2 wks after operation dur to sepsis	2200	M	live
Present case	1	spontaneously conceived	DCDA	23 + 3wks	cystic and solid	No	Yes	37 + 1wks	CS	2900 (SCT5.9 × 2.6 × 3.0 cm)	M	Underwent SCT resection at 4 days of age, **Live**	2880	M	live

*SCT* sacrococcygeal teratomas; *IVF* in vitro fertilization; *F *Female; *M *Male; *MCDA* monochorionic diamnionic; *DCDA* dichorionic diamniotic; *BW *Birth weight

Our case is the dichorionic diamniotic pregnancy and one twin had STC. During the pregnancy, both the maternal and fetal conditions were dynamically monitored, which was evaluated by multidisciplinary team. Due to no indications, we did not performed the for fetal intervention,Planned cesarean section was performed at full term. The prompt and successful surgical excision of the sacrococcygeal teratoma was performed and the condition of the SCT twin is good after operation. Both babies were followed up and are in good health until now.

Sacrococcygeal teratoma in twin pregnancy is rare. Early antenatal diagnosis is important. Once the sacrococcygeal teratoma is diagnosed, clinicians should be aware of the associated maternal and fetal complications. Expecting parents should be counseled by the multidisciplinary team about the management and prognosis of the STC twin and co-twin. Prompt surgical excision of the sacrococcygeal teratoma after birth should be suggested.

## Data Availability

The datasets used and/or analysed during the current study available from the corresponding author on reasonable request.

## List of Abbreviations

SCT: Sacrococcygeal teratoma
MRI: Magnetic Resonance Imaging
